# Ultrasonography for Injecting (Around) the Lateral Epicondyle: EURO-MUSCULUS/USPRM Perspective

**DOI:** 10.3390/diagnostics13040717

**Published:** 2023-02-14

**Authors:** Vincenzo Ricci, Kamal Mezian, Giulio Cocco, Giorgio Tamborrini, Giacomo Fari, Fiammetta Zunica, Ke-Vin Chang, Murat Kara, Levent Özçakar

**Affiliations:** 1 Physical and Rehabilitation Medicine Unit, Luigi Sacco University Hospital, ASST Fatebenefratelli-Sacco, 20157 Milan, Italy; 2 Department of Rehabilitation Medicine, First Faculty of Medicine and General University Hospital, Charles University, 12800 Prague, Czech Republic; 3 Unit of Ultrasound in Internal Medicine, Department of Medicine and Science of Aging, University “G. d’Annunzio”, 66100 Chieti, Italy; 4 UZR, Ultraschallzentrum und Institut für Rheumatologie, 4051 Basel, Switzerland; 5 Rheumatology Clinic, University Hospital of Basel, 4001 Basel, Switzerland; 6 Department of Translational Biomedicine and Neuroscience (DiBraiN), Aldo Moro University, 70121 Bari, Italy; 7 Department of Biological and Environmental Science and Technologies (Di.S.Te.B.A.), University of Salento, 73100 Lecce, Italy; 8 Department of Pediatrics, Vittore Buzzi Children’s Hospital, University of Milan, 20154 Milan, Italy; 9 Department of Physical Medicine and Rehabilitation, National Taiwan University Hospital, Bei-Hu Branch, Taipei 10845, Taiwan; 10 Department of Physical and Rehabilitation Medicine, Hacettepe University Medical School, 06100 Ankara, Turkey

**Keywords:** elbow, pain, tendon, ultrasonography, intervention

## Abstract

Lateral epicondylitis (LE) is a very common and painful condition seen in the daily practice of musculoskeletal physicians. Ultrasound-guided (USG) injections are commonly performed to manage the pain, promote the healing phase, and plan a tailored rehabilitation treatment. In this aspect, several techniques were described to target specific pain generators i the lateral elbow. Likewise, the aim of this manuscript was to extensively review those USG techniques together with the patients’ pertinent clinical/sonographic features. The authors believe that this literature summary could also be refined as a practical, ready-to-use guide for planning the USG interventions of the lateral elbow in clinical practice.

## 1. Introduction

Lateral epicondylitis (LE) can be considered as an umbrella definition—commonly used to include several pathological conditions involving the extra-articular soft tissues of the lateral elbow compartment [1]. Indeed, not only the common extensor tendon (CET), but also the subcutaneous tissue, the forearm fascia, and the tendon-bone junction (i.e., enthesis of the lateral epicondyle) can contribute to this clinical scenario [1]. Moreover, despite the historical “itis” in its name, it is well known that a very poor presence of white blood cells can be demonstrated within the soft tissues of the lateral elbow in relevant patients [2]. Instead, an intratendinous invasion of immature fibroblasts and chaotic neurovascular tangles were histologically identified, i.e., the so-called angiofibroblastic hyperplasia [3,4,5]. Epidemiologically, prospective studies and meta-analyses seem to strongly support the association between wrist/elbow biomechanics and the incidence of LE [6]. This “lateral overload” theory includes strenuous manual tasks such as a combination of repetitive movements and prolonged postures. It should also be kept in mind that the biological imbalance due to multiple microtraumas might exceed the healing responses of the soft tissues. 

Considering the multiple aforementioned (potential) pain generators and histopathological findings, several USG techniques were described/used to manage the lateral elbow pain as well as to plan for appropriate rehabilitation. Indeed, starting from the extremely variable sonographic findings encountered in patients with LE, a wide range of USG techniques can be used. Herewith, the pertinent literature also comprises incorrect and ‘historical’ terminology referring to those methods [7]. To the best of our knowledge, a comprehensive review of the technical aspects related to USG injections for LE is lacking; therefore, the authors—within the umbrella of EURO-MUSCULUS/USPRM—have summarized the different USG intervention techniques to inject soft tissues of the lateral elbow. For each type of intervention, technical notes, tips/tricks, and pitfalls were described to make the present manuscript also a practical guide for musculoskeletal physicians.

## 2. Materials and Methods

To develop a practical, ready-to-use guide for the management of LE patients in daily practice, the authors have planned a 3-step workflow as described below:

First phase: An extensive review of the scientific literature on USG injections for LE was performed, mainly focusing on different techniques used to inject soft tissues of the lateral elbow (rather than the biochemical nature of the injectate). PubMed and Web of Science were searched using the following keywords: “ultrasound”, “guided”, “injection”, “lateral” and “epicondylitis”.

Second phase: Different types of USG injections described in the literature were classified using an anatomical/histological approach. Accordingly, three main target zones were defined as the peritendinous area, the intratendinous area, and the attachment area of the tendon to the bony cortex of the lateral epicondyle.

Third phase: Different types of USG injections were matched with various sonographic findings characterizing the wide clinical spectrum of LE. Lastly, based on the clinical/sonographic experience of the authors, a few tips and tricks were proposed to accurately reach the anatomical target and to get better oriented for common procedural pitfalls.

## 3. Results and Discussion

Considering the special nature of the present review, the authors have combined the results and discussion sections to provide a unique layer-by-layer description of the different USG techniques for the lateral elbow. For each procedure (Table 1), the anatomical target and the related technical aspects were reported to optimize the clinical management in daily practice.

### 3.1. Peritendinous Injection

**Figure 1:**
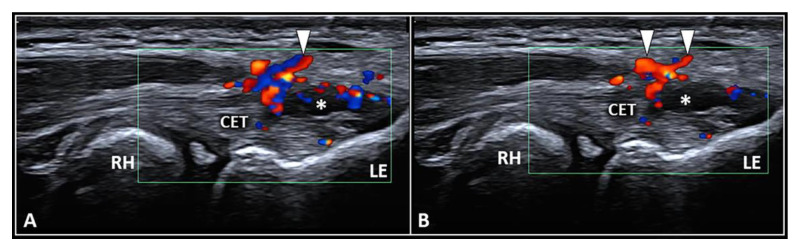
Ultrasound examination with color Doppler (**A**,**B**) clearly shows how the aberrant neovessels (*white arrowheads*) originating from the superficial vascular plexus of the subcutis pierce the forearm fascia invading the hypoechoic zone (*white asterisk*) of the CET related to insertional tendinosis. RH: radial head, LE: lateral epicondyle, CET: common extensor tendon.

The dermo-epidermal complex, subcutaneous tissue, and forearm fascia wrap the lateral elbow compartment and, using a high-frequency linear probe, they can all easily be identified as superficial to the CET [8,9]. At this level, the recurrent radial artery, posterior branch of the radial collateral artery, and the interosseous recurrent artery anastomose—forming a superficial vascular plexus which covers the lateral epicondyle and the CET [10]. Interestingly, in patients with signs and symptoms of LE, a hypertrophic neurovascular network was histologically identified within the aforementioned superficial soft tissues which envelops and penetrates the superficial fibers of the CET [11]. This condition is known as superficial-to-deep vascular invasion of the tendon tissue (Figure 1)—with larger vascular elements being in the subcutaneous tissue (i.e., the donator vessels) and thinner vascular signals residing within the CET (i.e., the penetrating vessels) [1]. 

To optimize the sonographic visibility of the aforementioned vascular infiltration of the CET, the authors suggest a few technical tips and tricks. At first, a large amount of gel should be used, i.e., the suspension technique—to avoid/minimize the compression of skin with the ultrasound probe as well as the unintentional squeeze of the small-sized vascular elements [8,9]. Second, by adjusting the frame rate and positioning the superior edge of the color/power Doppler box at the level of the subcutaneous tissue, correct assessment of the superficial microvasculature can be performed—avoiding fake vascular signals related to acoustic artifacts [12]. Lastly, a large region of interest should be avoided to preserve the Doppler sensitivity [12].

In the authors’ experience, a frequent inaccuracy (especially for beginners) during the sonographic assessment of tendon vascularization is incomplete scanning, i.e., focusing on the distal portion and ignoring its most proximal section. In this sense, we strongly suggest fully scrutinizing the CET by shifting the probe proximally until the sonographic visualization of the extensor carpi radialis longus muscle, which directly attaches to the humerus without a tendinous component, i.e., a muscle-bone interface [13]. 

Based on the anatomical and histopathological findings quoted above, USG injection of the interface between the forearm fascia and the underlying CET can be performed to disrupt the bridging neovessels and neonerves originating from the superficial tissues and infiltrating the tendon fibers [1]. An in-plane technique is recommended to guarantee real-time visualization of the needle for its entire course during the intervention and a proximal-to-distal or distal-to-proximal approach can be used according to the physician’s experience [14]. 

Especially for beginners, the main pitfall of the USG peritendinous injection of the CET would be the risk of advancing the needle’s tip within the most superficial fibers of the tendon and not at the extra-tendinous fascial layers. In this aspect, the authors suggest a few tips/tricks to avoid the aforementioned complication [15]. 

**Figure 2:**
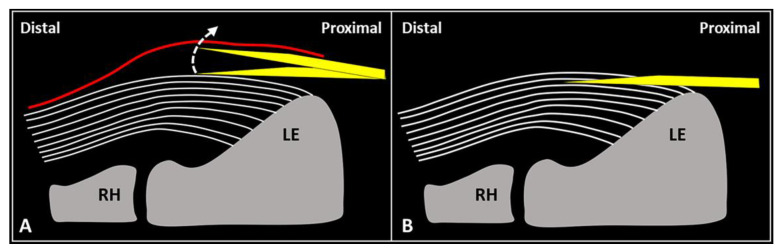
When the needle’s tip (*yellow*) is correctly positioned just over the superficial edge of the CET, by gently tilting (*curved dotted arrow*) the needle, it is possible to “separate” the forearm fascia (*red*) from the underlying tendon without hard resistance (**A**). Conversely, if the needle’s tip (*yellow*) is located inside the superficial portion of the CET, a “block” can be felt during this maneuver due to the intratendinous entrapment of the needle between the tendon fibers (*white lines*) (**B**). RH: radial head, LE: lateral epicondyle.

First, gentle inclination of the needle pointing the tip toward the skin can be performed. The movement can be easily performed when the needle is located inside the soft superficial tissues, while a stiff “block” can be encountered as the needle is inserted within the tendon (Figure 2). Second, slight pressure can be applied on the syringe plunger to observe if the mixture can be easily injected or not. However, hard resistance can be felt if the needle is located within the tendon fibers and very low resistance (coupled with smooth flow of the mixture covering the tendon surface) can be observed when the needle tip is positioned inside the fascial layers.

Since mechanical detachment of the forearm fascia/subcutaneous tissue from the underlying CET is the main goal of this procedure, i.e., to release eventual adhesions related to chronic frictions and to disrupt the perforating neovessels/neonerves, extra attention should be paid to the volume injected. Herein, the authors suggest to use at least 4 mL of volume to efficiently perform hydrodissection of the tendon-fascia interface. 

In patients with lateral elbow tendinopathy, Pellegrino et al. [16] have recently demonstrated that the combination of a USG peritendinous injection with hyaluronic acid and high-intensity laser therapy (compared to therapeutic exercise) might be more effective for improving pain, muscle strength, and disability at a 1 to 3 month follow-up. They used a 25 G, 25 mm long needle, and 2 mL of a pre-filled syringe with 20 mg of linear hyaluronic acid with a molecular weight of 500–730 kDa without using local anesthetic [16]. An in-plane technique, proximal-to-distal approach was preferred by the authors to advance the needle’s tip within the peritendinous area of the lateral epicondyle [16]. The authors stated that both the pharmacological effects of the hyaluronic acid and the lubricating effect reducing tendon rubbing in the pre-insertional zone played a pivotal role in the clinical and functional outcomes observed in their retrospective study [16]. 

Fogli et al. [17] performed three USG peritendinous injections (1/week) with 2 mL of hyaluronic acid (molecular weight = 500–730 kDa) in 26 lateral elbow tendinopathies with follow-ups at 7, 14, and 56 days after the first injection. Pain, CET thickness and intratendinous vascularity decreased significantly at each endpoint [17]. To this end, the tendon-fascia interface hydrodissection seems to effectively reduce the vascular invasion of the CET by the superficial neurovascular plexus of the soft tissues [1,10,11].

### 3.2. Intratendinous Injection

**Figure 3:**
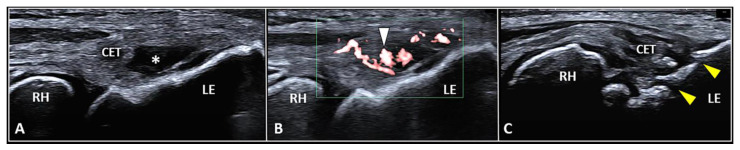
Partial tear (*white asterisk*) of the insertion zone of CET (**A**), focal tendinosis with inner hypervascularization (*white arrowhead*) (**B**), and enthesitis with cortical irregularities (*yellow arrowhead*) (**C**) of the lateral epicondyle (*LE*) should be considered among the most common sonographic findings related to the clinical picture of lateral epicondylitis. RH: radial head.

The CET is an anchor tendon, mainly composed of longitudinally oriented collagen fibers attaching to the lateral epicondyle of the humerus. Normally, it presents a hyperechoic fibrillar pattern quite similar to the underlying RCL [1]. The extensor carpi radialis brevis tendon represents its deepest layer whereas the extensor digitorum communis tendon constitutes the most superficial portion of the common tendinous mass [18]. Interestingly, extensor carpi radialis longus, extensor carpi ulnaris, and the CET are not fused with each other—presenting as separate anatomical structures [19]. The vascular network is not uniformly distributed within the CET and unlike the superficial layers perfused by a rich subcutaneous plexus, the deep portion is almost avascular [10]. Among several pathological conditions potentially involved in the clinical scenario of LE, focal tendinosis, partial tear, and intratendinous calcific deposition are the most commonly encountered in daily practice (Figure 3) [1]. 

For all the aforementioned disorders, color/power Doppler assessment should be coupled with the B-mode to visualize the presence of aberrant neovessels within the pathological tendon tissue [1,3,4]. Zeising et al. demonstrated that USG injection of the intratendinous microvasculature with local anesthetic efficiently reduced lateral elbow pain, suggesting that only the pathological (neuro)vascular tangle could represent the main pain generator in patients with LE [4]. Interestingly, using sclerosing polidocanol instead of local anesthetic, they described several cases characterized by a significant pain reduction despite the persistence of intratendinous hypervascularization on color/power Doppler [20]. In this sense, it seems that the neonerves more than the neovessels play a pivotal role in painful tendinopathy of the LE. Based on speculations from the aforementioned findings, the authors used the vascular signals of color/power Doppler as an indirect sonographic sign to localize the real target of procedure, i.e., the intratendinous neural tangle [20]. More specifically, Substance P and calcitonin gene-regulated peptide seem to be the main neuropeptides involved in (i) neurogenic inflammation of tendon sensory fibers and (ii) microvascular leakage with local edema [21]. 

Using B-mode (tendon thickness, bony spur) and color Doppler (vascular signals), Krogh et al. [22] published a cross-sectional study of 264 participants with healthy elbows and 60 patients with chronic LE. Interestingly, they reported that color doppler activity was a strong indicator of ongoing tendinopathy (although not pathognomonic) and that the absence of vascular signals in patients with suspected lateral tendinopathy should raise the suspicion of an alternative diagnosis [22]. Likewise, in a between-group cross-sectional study of 25 patients and 19 asymptomatic participants, du Toit et al. [23] concluded that neovascularity identified with power Doppler ultrasonography (vs. B-mode imaging) was diagnostically superior in identifying chronic tennis elbow. Moreover, the lack of both neovascularity and grey scale changes in ultrasound examinations substantially increased the probability that the condition is not present and should prompt the clinician to consider other causes for lateral elbow pain [23]. 

Recently, dynamic imaging protocols of the elbow augmented the diagnostic potential of ultrasound examination in detecting soft tissue injuries which could hardly be identified. For instance, in patients with signs/symptoms suggestive of LE, dynamic assessment can be performed with active/passive movements of the elbow—to use the intraarticular synovial fluid as a natural contrast agent, pushing it within a partial tear of the CET on its articular side [24]. Interestingly, in some patients, a painful pinching of the radio-humeral synovial plica can be observed between the bony surfaces, which can otherwise mimic the painful/clinical scenario of LE [24]. Especially in young athletes with chronic lateral elbow pain refractory to first-line treatments, we suggest performing a dynamic ultrasound assessment to evaluate challenging pathologies, such as the proximal radioulnar joint instability [24]. Indeed, repetitive mechanical stress can progressively induce a micro-elongation of the annular ligament complex with hypermobility of the radial head, leading to local synovitis and chondral lesions.

Considering the superficial location of the CET, a 25 to 38 mm needle is considered long enough to accurately reach the tendon tissue in the literature [25]. Likewise, 18–25 G needles can be used depending on the technical features of the procedure. Of note, a thinner needle can be used to inject an orthobiologic agent within a partial tear, a larger needle is necessary to perform USG fenestrations in focal tendinosis (Figure 3). As previously mentioned for peritendinous injections, an in-plane technique is also recommended to visualize the needle’s shaft and tip during intratendinous procedures [14]. Again, a proximal-to-distal or distal-to-proximal approach can be used. Needless to say, to plan for an accurate injection, the authors suggest matching the longitudinal and transverse scans to exactly “pinpoint” the pathological segment of the common tendinous mass [26,27]. 

**Figure 4:**
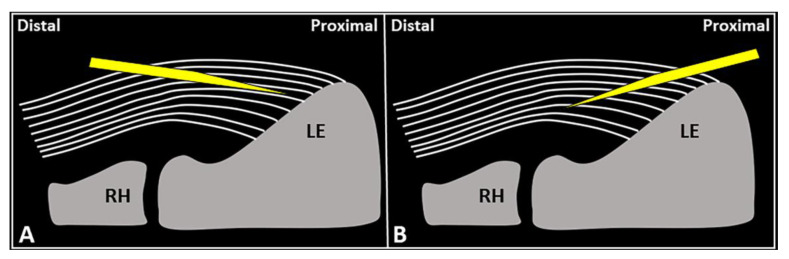
Schematic drawing shows the distal-to-proximal (**A**) and proximal-to-distal (**B**) approaches for performing USG fenestration of the CET. While the needle tip (*yellow*) can slip in between the tendon fibers (*white lines*) in the former technique (**A**); crossing in an orthogonal fashion, it efficiently disrupts them in the latter (**B**). RH: radial head, LE: lateral epicondyle.

In our experience, we suggest using a specific approach depending on the main purpose of the intervention:

***USG injection of the CET***: a distal-to-proximal approach (Figure 4) allows for reaching a very specific zone of the CET, especially if located in close proximity to the attachment zone, e.g., a focal disruption of the fibers near the fibrocartilaginous plate of the enthesis. Of note, several authors in the pertinent literature have clearly demonstrated that the volume injected within the CET partially migrates outside, reaching the surrounding tissues of the lateral elbow compartment. Park et al. [28] performed a USG intratendinous injection of 1.5 mL of platelet-rich plasma into the CET of 25 patients with chronic LE (duration of symptoms > 6 months). They evaluated the spread of the mixture immediately after the procedure. Interestingly, the mixture was sonographically identified as fluids and/or gas microbubbles both inside the subcutaneous tissue and within the radio-humeral joint [28]. In this sense, the CET should not be considered as a sealed chamber but rather as a multilayer structure in which fluid can slip between the interlaminar zones and leak out from the tendon structure. 

***USG fenestration of the CET***: a proximal-to-distal approach might guarantee needle movements orthogonal to the spatial orientation of the CET fibers (Figure 4). The bevel of the needle can efficiently disrupt the tendon tissue, i.e., the main goal of USG fenestration. Instead, if a distal-to-proximal approach is used, the needle tends to slip in between the different layers of the CET—traveling inside the interlaminar zones without a satisfactory transection of the fibers (Figure 4). The exact number of back-and-forward passages of the needle within the CET is not well defined in the literature, whereby the feeling of local softness is usually considered as practical clinical feedback for a successful procedure. In addition, a small amount of blood might commonly flow back to the syringe—due to mechanical disruption of the aforementioned abundant microvasculature of the CET in pathological conditions.

Stenhouse et al. [29] published a prospective, randomized pilot study involving 28 patients with refractory LE with a mean symptom duration of 19.1 months. In 13 patients, USG fenestration alone was performed, whereas in 15 patients it was also combined with a USG injection of autologous conditioned plasma. Both groups received two procedures at 0 weeks and at 1 month, with a two-step follow-up at 2 months and 6 months. Interestingly, visual analogue pain and Nirschl scores were observed to be similar between the two groups at each follow-up interval [29]. Likewise, with an average follow-up of 22 months, McShane et al. [30] demonstrated good or excellent outcomes in 92.3% of patients who underwent USG fenestration of the CET as a unique interventional procedure for recalcitrant LE. 

Based on the currently available evidence, it seems that the mechanical effects of the USG procedure—rather than the chemical effects of the injectate—may play a more pivotal role in the clinical/functional outcomes. Indeed, after repetitive perforation of a tendon, transcription factors involved in the proliferation and differentiation of mesenchymal cells, genes involved in inflammatory responses, and angiogenesis pathways are regulated, modulating the healing response (Supplementary Figure S1) [31,32,33]. Histologically, the back-and-forward movements of the needle within the pathological tendon tissue induce micro-injuries and local bleeding with progressive deposition of granulation tissue that mechanically reinforces/stabilizes the tendon microarchitecture [32,34].

**Figure 5:**
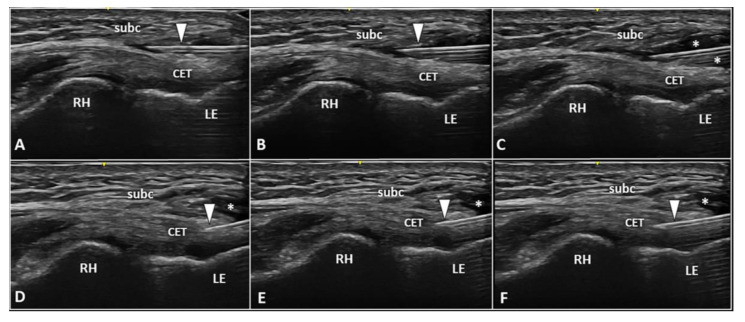
Two-step USG procedure of the lateral elbow using in-plane proximal-to-distal approach. The needle (*white arrowhead*) is advanced in between the subcutaneous tissue (*subc*) and the CET. High volume (*white asterisks*) is injected to efficiently “open” the pathological interface (**A**–**C**). The needle (*white arrowhead*) is redirected more deeply to perform the fenestration of the pre-insertional segment of CET (**D**–**F**). RH: radial head, LE: lateral epicondyle.

Notably, different USG techniques can be combined if clinically indicated. For instance, in a patient with sonographic findings of a superficial-to-deep vascular invasion of pre-insertional focal tendinosis of CET, a two-step procedure can be planned. First, a high-volume USG hydrodissection of the tendon-fascia interface can be performed to release adhesions and to disrupt the perforating neovessels/neonerves. Second, a USG fenestration of the degenerated tendon tissue can be performed to transect the disorganized fibers with multiple back-and-forward movements of the needle (Figure 5). 

Recently, Pringels et al. [35] theorized that the remodeling of tendon tissue into fibrocartilage-like tissue can cause increased intratendinous resting pressure, mainly due to excessive water-binding glycosaminoglycans and proteoglycans. Moreover, this increased pressure might explain the hypoxic state and the formation of leaky (neo)vessels in tendon pathology [35]. Based on this very innovative and interesting conceptual framework, we speculate that clinical outcomes related to the USG fenestration of the CET may be partially related to the decompressive effect of the procedure. In this sense, repetitive passages of the needle through the tendon can promote spilling of extra fluids from the intra and interfascicular compartments.

### 3.3. Enthesis Injection

The fibers of the CET anchor to the lateral epicondyle through a transitional plate known as enthesis. Histologically, it is a double-layer structure made of an uncalcified fibrocartilaginous stratum in continuum with the tendon fibers and a calcified fibrocartilage attached to the subchondral bone [36,37]. In physiological conditions, the sonographic appearance of lateral enthesis is similar to a smooth hypoechoic band located between the hyperechoic cortical surface of the lateral epicondyle and the regular fibrillar pattern of the CET. [1,38,39] Likewise, in patients with enthesopathies, focal interruptions of the cortical bone, pitting of the trabecular bone, lamellar calcifications of the fibrocartilaginous plate, and vascular signals at color/power Doppler are the most common sonographic findings (Figure 3) [1,38,39].

Several authors have proposed USG procedures focused on the uncalcified and/or calcified layer of the tendon-bone junction. McShane et al. [40] have described a USG intervention directed to the lateral elbow enthesis in patients with chronic/refractory tendinosis of the CET. Using an in-plane distal-to-proximal approach, the needle’s tip was used to abrade the periosteum of the lateral epicondyle, scraping the bony edge [40].

Yoo et al. [41] a two-step USG procedure in a cluster of LE patients without a response to nonsurgical treatments. First, a USG distension of the focal tendon injury at the tendon-bone interface was performed using a saline solution, “exposing” the underlying enthesis. Then, a USG drilling of the bone underlying the focal tendon tear was performed with an electric drill [41]. Histologically, decortication of the lateral epicondyle enhances blood supply to the devitalized tendon tissue promoting the healing process [42]. Interestingly, at 1, 3, and 6 month follow ups, visual analogue scale, maximum voluntary grip strength, and the patient-related tennis elbow evaluation score improved with progressive/sonographic reduction in the lesion size. 

**Figure 6:**
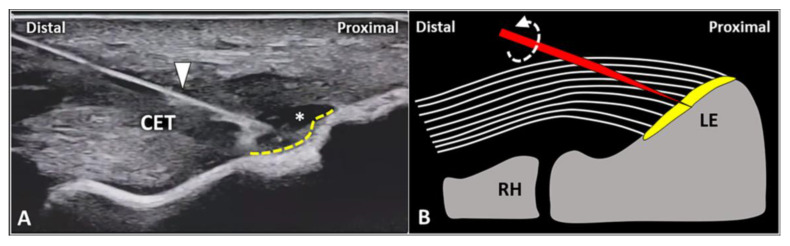
In a patient with focal avulsion of deep fibers of CET from the lateral epicondyle (*LE*), a two-step USG procedure can be performed. Using an in-plane distal-to-proximal approach, the needle (*white arrowhead*) is advanced within the injury (*white asterisk*) and local anesthetic is injected to “uncover” the underlying enthesis (*yellow dotted line*) (**A**). Schematic drawing shows multiple rotations (*curved dotted arrow*) of the needle’s tip (*red*) for mechanical drilling of the fibrocartilage (*yellow*) to induce a local micro-bleeding (**B**). RH: radial head.

In the authors’ experience, the USG injection of the enthesis is less commonly performed when compared to peri/intratendinous injections in the daily management of patients with LE. Likewise, focal detachment (i.e., avulsion) of the CET fibers from the fibrocartilaginous plate of the lateral elbow can be considered a non-rare pathological condition requiring this intervention. Technically, using the in-plane distal-to-proximal approach, we suggest a two-step procedure (Figure 6) with an intralesional injection of local anesthetic followed by abrasion of the fibrocartilage with rotations of the needle bevel causing a “drilling effect”.

Of note, not rarely in daily practice, radio-humeral synovitis can mimic the clinical scenario of lateral enthesopathy [43]. Intra-articular effusion in the annular recess and vascular signals within/around the radio-humeral synovial plica are the most common sonographic findings suggestive of articular pain [1,12]. Therefore, a USG intra-articular rather than an extra-articular procedure should be planned for prompt management [44]. With the elbow flexed at 90 degrees and the forearm pronated, an out-of-plane technique—by advancing the needle through the CET—is usually preferred to release the mixture inside the synovial space of the elbow [45]. A hyperechoic flash within the hypo/anechoic radio-humeral cleft during the USG procedure can be considered as a common “confirming” sign for correct intra-articular placement.

Chen et al. [46] have described a two-step USG procedure to simultaneously inject the radio-humeral joint and the tendon-bone interface of the CET. Using an out-of-plane technique with a lateral approach, the white dot representing the cross-section of the needle is first within the articular cleft and subsequently inside the enthesis of the lateral epicondyle. As such, the authors have considered the lateral elbow pain (refractory to conservative treatments) as cross-talking between two different pain generators—the fibrocartilaginous plate of the CET and the synovial tissue of the radio-humeral joint. 

Lastly, among the different types of USG techniques described in the pertinent literature to manage chronic pain in patients with recalcitrant LE, muscular injections have also been reported. Indeed, considering the excessive traction of the muscle-tendon unit, a well-established pathophysiological element for progression to the chronic phase of lateral epicondylopathy, botulinum toxin injections into the forearm extensor muscles were performed [47]. Of note, very careful selection of the muscle(s) to be injected and the dosage of toxin for each muscle belly have to be planned before the intervention to avoid adverse events such as muscular weakness [48]. 

## 4. Conclusions

Lateral epicondylitis (LE) can be considered as a non-specific definition related to several pathological conditions involving soft tissues of the lateral elbow. Being aware of the mounting role of US examinations in decoding the (widely variable) underlying (painful) etiologies of this clinical scenario, a corresponding range of different USG injections needs to be on the agenda of musculoskeletal physicians. In other words, the use of US is crucial; first, for better understanding the pathology (and thus for better clinical decision making as regards the interventional procedure); second, it provides precise targeting/guidance during the onward procedure which is tailored according to each/every patient [49]. As an international group of experts in musculoskeletal ultrasound, and under the umbrella of EURO-MUSCULUS/USPRM, we have proposed the present *pain generator-based approach* as a practical, ready-to-use guide in clinical practice. Last but not least, the authors also draw attention to the lack of uniform terminology and robust outcome data as far as these USG procedures are concerned.

## Figures and Tables

**Table 1:** USG injections for Lateral Epicondylitis.

Procedure	Target	Technical Note
Peritendinous	Interface between the CET and the forearm fascia	High-volume hydrodissection detaching the fascia from the underlying CET *
Intratendinous	Inside a specific portion of the CET	Intralesional injection and/or fenestration of the degenerated area
Enthesis	Tendon-bone interface	Intralesional injection and/or fibrocartilage plate abrasion

* Release of the adhesions and disruption of the bridging neovessels/nerves. CET: common extensor tendon.

## Data Availability

Not applicable.

## Supplementary Materials

The following supporting information can be downloaded at: https://www.mdpi.com/article/10.3390/diagnostics13040717/s1, Supplementary Figure S1: Biological effects of USG fenestration of CET. TNF: tumor necrosis factor, IL: interleukin, VEGF: vascular endothelial growth factor, MMP: matrix metalloproteinase.

## Abbreviation

**EURO-MUSCULUS/USPRM**	European Musculoskeletal Ultrasound Study Group/Ultrasound Study Group of the International Society of Physical and Rehabilitation Medicine

## References

[1] Ricci V., Cocco G., Mezian K., Chang K.V., Naňka O., Tamborrini G., Kara M., Özçakar L. (2022). Anatomy and sonographic examination for lateral epicondylitis: EURO-MUSCULUS/USPRM* approach. Am. J. Phys. Med. Rehabil..

[2] Binder A.I., Hazleman B.L. (1983). Lateral humeral epicondylitis–a study of natural history and the effect of conservative therapy. Br. J. Rheumatol..

[3] Nirschl R.P., Ashman E.S. (2003). Elbow tendinopathy: Tennis elbow. Clin. Sports Med..

[4] Zeisig E., Ohberg L., Alfredson H. (2006). Extensor origin vascularity related to pain in patients with Tennis elbow. Knee Surg. Sports Traumatol. Arthrosc..

[5] Potter H.G., Hannafin J.A., Morwessel R.M., DiCarlo E.F., O’Brien S.J., Altchek D.W. (1995). Lateral epicondylitis: Correlation of MR imaging, surgical, and histopathologic findings. Radiology.

[6] Descatha A., Albo F., Leclerc A., Carton M., Godeau D., Roquelaure Y., Petit A., Aublet-Cuvelier A. (2016). Lateral epicondylitis and physical exposure at work? A review of prospective studies and meta-analysis. Arthritis Care Res..

[7] Hall M.M., Allen G.M., Allison S., Craig J., DeAngelis J.P., Delzell P.B., Finnoff J.T., Frank R.M., Gupta A., Hoffman D. (2022). Recommended musculoskeletal and sports ultrasound terminology: A delphi-based consensus statement. Br. J. Sports Med..

[8] Ricci V., Ricci C., Cocco G., Donati D., Farì G., Mezian K., Naňka O., Özçakar L. (2022). From histology to sonography in skin and superficial tissue disorders: EURO-MUSCULUS/USPRM* approach. Pathol. Res. Pract..

[9] Ricci V., Ricci C., Gervasoni F., Andreoli A., Özçakar L. (2022). From histo-anatomy to sonography in lymphedema: EURO-MUSCULUS/USPRM approach. Eur. J. Phys. Rehabil. Med..

[10] Schneeberger A.G., Masquelet A.C. (2002). Arterial vascularization of the proximal extensor carpi radialis brevis tendon. Clin. Orthop. Relat. Res..

[11] Spang C., Alfredson H. (2017). Richly innervated soft tissues covering the superficial aspect of the extensor origin in patients with chronic painful tennis elbow—Implication for treatment?. J. Musculoskelet. Neuronal Interact..

[12] Ricci V., Ricci C., Tamborrini G., Chang K.V., Mezian K., Zunica F., Naňka O., Kara M., Özçakar L. (2023). From histology to sonography in synovitis: EURO-MUSCULUS/USPRM approach. Pathol. Res. Pract..

[13] Omoumi P., Gondim Teixeira P.A., Ward S.R., Trudell D., Resnick D. (2021). Practical ultrasonographic technique to precisely identify and differentiate tendons and ligaments of the elbow at the level of the humeral epicondyles: Anatomical study. Skelet. Radiol..

[14] Ricci V., Abdulsalam A.J., Özçakar L. (2019). Ultrasound Imaging for Dummies: Getting oriented among the planes. J. Rehabil. Med..

[15] Ricci V., Özçakar L. (2020). From “ultrasound imaging” to “ultrasound examination”: A needful upgrade in musculoskeletal medicine. Pain Med..

[16] Pellegrino R., Paolucci T., Brindisino F., Mondardini P., Di Iorio A., Moretti A., Iolascon G. (2022). Effectiveness of high-intensity laser therapy plus ultrasound-guided peritendinous hyaluronic acid compared to therapeutic exercise for patients with lateral elbow tendinopathy. J. Clin. Med..

[17] Fogli M., Giordan N., Mazzoni G. (2017). Efficacy and safety of hyaluronic acid (500–730 kDa) ultrasound-guided injections on painful tendinopathies: A prospective, open label, clinical study. Muscles Ligaments Tendons J..

[18] Clarke A.W., Ahmad M., Curtis M., Connell D.A. (2010). Lateral elbow tendinopathy: Correlation of ultrasound findings with pain and functional disability. Am. J. Sports Med..

[19] Greenbaum B., Itamura J., Vangsness C.T., Tibone J., Atkinson R. (1999). Extensor carpi radialis brevis. An anatomical analysis of its origin. J. Bone Jt. Surg. Br..

[20] Zeisig E., Ohberg L., Alfredson H. (2006). Sclerosing polidocanol injections in chronic painful tennis elbow-promising results in a pilot study. Knee Surg. Sports Traumatol. Arthrosc..

[21] Ljung B.O., Forsgren S., Fridén J. (1999). Substance P and calcitonin gene-related peptide expression at the extensor carpi radialis brevis muscle origin: Implications for the etiology of tennis elbow. J. Orthop. Res..

[22] Krogh T.P., Fredberg U., Ammitzbøll C., Ellingsen T. (2020). Clinical value of ultrasonographic assessment in lateral epicondylitis versus asymptomatic healthy controls. Am. J. Sports Med..

[23] du Toit C., Stieler M., Saunders R., Bisset L., Vicenzino B. (2008). Diagnostic accuracy of power doppler ultrasound in patients with chronic tennis elbow. Br. J. Sports Med..

[24] Ricci V., Güvener O., Chang K.V., Wu W.T., Mezian K., Kara M., Leblebicioğlu G., Pirri C., Ata A.M., Dughbaj M. (2022). EURO-MUSCULUS/USPRM dynamic ultrasound protocols for elbow. Am. J. Phys. Med. Rehabil..

[25] Sussman W.I., Williams C.J., Mautner K. (2016). Ultrasound-guided elbow procedures. Phys. Med. Rehabil. Clin. N. Am..

[26] Ricci V., Schroeder A., Özçakar L. (2020). Ultrasound imaging for lateral elbow pain: Pinpointing the epicondylosis. Am. J. Phys. Med. Rehabil..

[27] De Maeseneer M., Brigido M.K., Antic M., Lenchik L., Milants A., Vereecke E., Jager T., Shahabpour M. (2015). Ultrasound of the elbow with emphasis on detailed assessment of ligaments, tendons, and nerves. Eur. J. Radiol..

[28] Park G.Y., Kwon D.R., Cho H.K., Park J., Park J.H. (2017). Distribution of platelet-rich plasma after ultrasound-guided injection for chronic elbow tendinopathies. J. Sports Sci. Med..

[29] Stenhouse G., Sookur P., Watson M. (2013). Do blood growth factors offer additional benefit in refractory lateral epicondylitis? A prospective, randomized pilot trial of dry needling as a stand-alone procedure versus dry needling and autologous conditioned plasma. Skelet. Radiol..

[30] McShane J.M., Shah V.N., Nazarian L.N. (2008). Sonographically guided percutaneous needle tenotomy for treatment of common extensor tendinosis in the elbow: Is a corticosteroid necessary?. J. Ultrasound Med..

[31] Hammerman M., Aspenberg P., Eliasson P. (2014). Microtrauma stimulates rat achilles tendon healing via an early gene expression pattern similar to mechanical loading. J. Appl. Physiol. (1985).

[32] Darrieutort-Laffite C., Soslowsky L.J., Le Goff B. (2020). Molecular and structural effects of percutaneous interventions in chronic achilles tendinopathy. Int. J. Mol. Sci..

[33] Calderón-Díez L., Sánchez-Sánchez J.L., Herrero-Turrión J., Cleland J., Arias-Buría J.L., Fernández-de-Las-Peñas C. (2021). Dry needling of a healthy rat achilles tendon increases its gene expressions: A pilot study. Pain Med..

[34] Stoychev V., Finestone A.S., Kalichman L. (2020). Dry needling as a treatment modality for tendinopathy: A narrative review. Curr. Rev. Musculoskelet. Med..

[35] Pringels L., Cook J.L., Witvrouw E., Burssens A., Vanden Bossche L., Wezenbeek E. (2022). Exploring the role of intratendinous pressure in the pathogenesis of tendon pathology: A narrative review and conceptual framework. Br. J. Sports Med..

[36] Milz S., Tischer T., Buettner A., Schieker M., Maier M., Redman S., Emery P., McGonagle D., Benjamin M. (2004). Molecular composition and pathology of entheses on the medial and lateral epicondyles of the humerus: A structural basis for epicondylitis. Ann. Rheum. Dis..

[37] Kehl A.S., Corr M., Weisman M.H. (2016). Review: Enthesitis: New insights into pathogenesis, diagnostic modalities, and treatment. Arthritis Rheumatol..

[38] Tamborrini G., Bruyn G.A. (2020). CME-Sonografie 93: Ultraschall der Enthese—Nicht jede «Enthesitis» bedeutet eine Spondyloarthritis [CME Sonography 93: Ultrasound of the Enthesis—Not Every “Enthesitis” Signals a Spondyloarthritis]. Praxis.

[39] Gandjbakhch F., Terslev L., Joshua F., Wakefield R.J., Naredo E., D’Agostino M.A., OMERACT Ultrasound Task Force (2011). Ultrasound in the evaluation of enthesitis: Status and perspectives. Arthritis Res. Ther..

[40] McShane J.M., Nazarian L.N., Harwood M.I. (2006). Sonographically guided percutaneous needle tenotomy for treatment of common extensor tendinosis in the elbow. J. Ultrasound Med..

[41] Yoo S.H., Cha J.G., Lee B.R. (2018). Ultrasound-guided percutaneous bone drilling for the treatment of lateral epicondylitis. Eur. Radiol..

[42] Ahmad Z., Siddiqui N., Malik S.S., Abdus-Samee M., Tytherleigh-Strong G., Rushton N. (2013). Lateral epicondylitis: A review of pathology and management. Bone Jt. J..

[43] Ricci V., Becciolini M., Özçakar L. (2020). Ultrasound imaging for recalcitrant lateral elbow pain: Radio-humeral synovial plica is also at play. Pain Med..

[44] Ricci V., Özçakar L. (2020). Ultrasound-guided intra-articular injection of the elbow: Targeting deep to anconeus muscle. Pain Med..

[45] Tortora S., Messina C., Albano D., Serpi F., Corazza A., Carrafiello G., Sconfienza L.M., Gitto S. (2021). Ultrasound-guided musculoskeletal interventional procedures around the elbow, hand and wrist excluding carpal tunnel procedures. J. Ultrason..

[46] Chen J.L., Cheng W.J., Chen K.Y., Chen C.P.C. (2022). Ultrasound-guided injection approach of treating tennis elbow and elbow joint synovitis simultaneously under one needle insertion point. Am. J. Phys. Med. Rehabil..

[47] Kalichman L., Bannuru R.R., Severin M., Harvey W. (2011). Injection of botulinum toxin for treatment of chronic lateral epicondylitis: Systematic review and meta-analysis. Semin. Arthritis Rheum..

[48] Galván Ruiz A., Vergara Díaz G., Rendón Fernández B., Echevarría Ruiz De Vargas C. (2019). Effects of ultrasound-guided administration of botulinum toxin (incobotulinumtoxin A) in patients with lateral epicondylitis. Toxins.

[49] Ricci V., Özçakar L. (2019). Life after ultrasound: Are we speaking the same (or a new) language in physical and rehabilitation medicine?. J. Rehabil. Med..

