# Supplementary figures and images for: Ultrasonography for Injecting (Around) the Lateral Epicondyle: EURO-MUSCULUS/USPRM Perspective
Source: Diagnostics (Basel). 2023 Feb 14;13(4):717. doi: 10.3390/diagnostics13040717 (PMC9955720; doi:10.3390/diagnostics13040717)

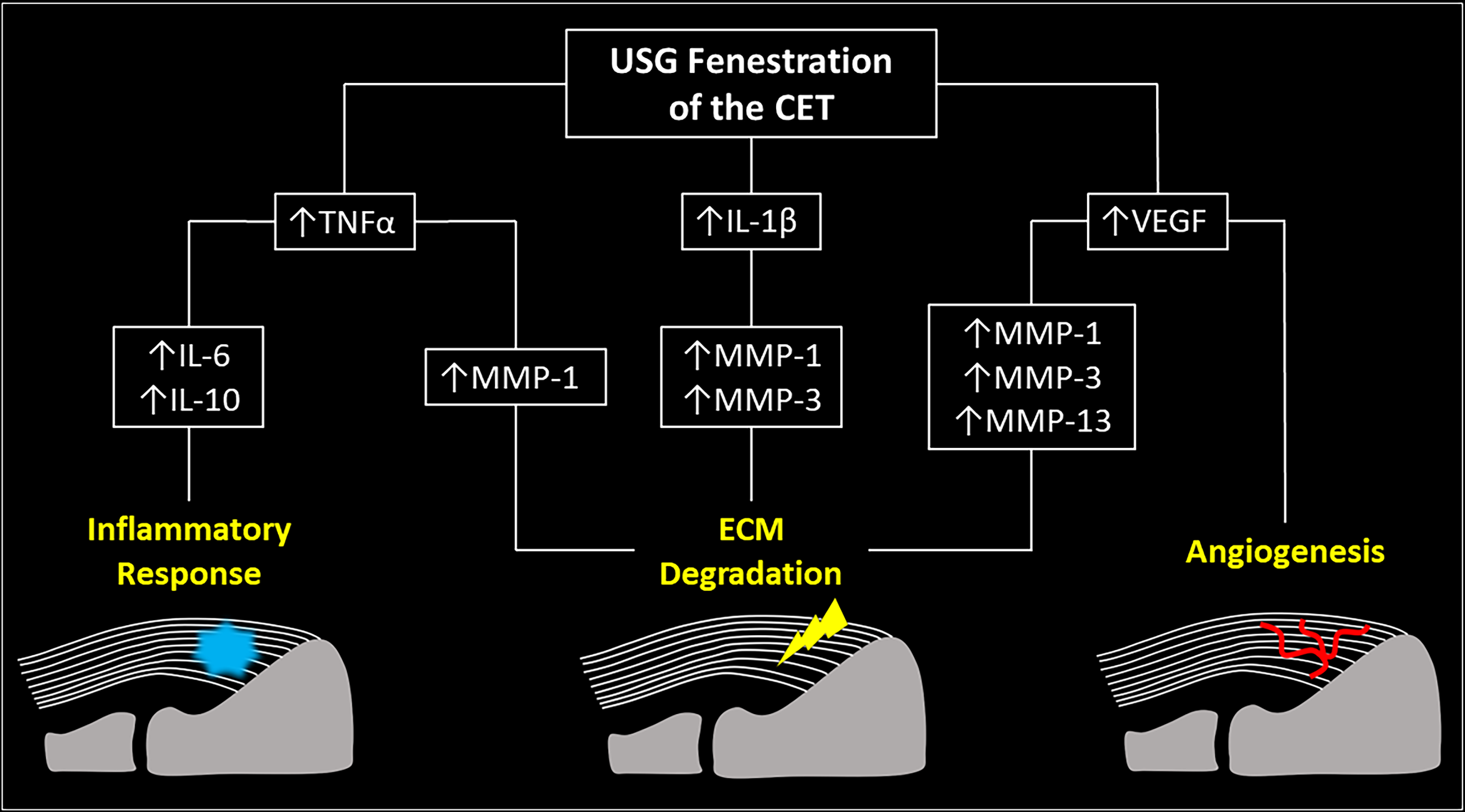

Supplement: Supplementary file 1 [file diagnostics-13-00717-s001.zip › Figure S1 Biological effects of USG fenestration of CET. TNF tumor necrosis factor, IL interleu-kin, VEGF vascular endothelial growth factor, MMP matrix metalloproteinase.tif]
